# Ubiquitin-Like Protein UBD Promotes Cell Proliferation in Colorectal Cancer by Facilitating p53 Degradation

**DOI:** 10.3389/fonc.2021.691347

**Published:** 2021-07-14

**Authors:** Hongbin Su, Mengdi Qin, Qiang Liu, Bo Jin, Xianjun Shi, Zheng Xiang

**Affiliations:** ^1^ Department of Gastrointestinal Surgery, The First Affiliated Hospital of Chongqing Medical University, Chongqing, China; ^2^ Chongqing Key Laboratory of Department of General Surgery, The First Affiliated Hospital of Chongqing Medical University, Chongqing, China

**Keywords:** colorectal cancer, UBD, p53, proliferation, degradation

## Abstract

**Purpose:**

Ubiquitin D (UBD) is a member of the ubiquitin-like modifier (UBL) family and is highly expressed in a variety of cancers including colorectal cancer (CRC). However, the mechanisms of its regulatory roles in CRC are largely elusive. In this study, we revealed the effect of UBD on the proliferation of CRC.

**Methods:**

The expression of UBD in clinical tissue samples of CRC and seven CRC cell lines was detected using qRT-PCR, immunohistochemistry (IHC) and Western blotting. CCK-8, colony formation, EdU and flow cytometry assays were used to detect the functional changes of CRC cells transfected with UBD stable expression plasmids *in vitro*. A xenograft model was constructed to assess the effect of UBD on the growth of CRC cells *in vivo*. The connection between UBD and p53 was analyzed using Western blotting, immunoprecipitation, proteasome inhibition assay and Cycloheximide (CHX) chase assay.

**Results:**

UBD was overexpressed in CRC tumor tissues compared with nontumor tissues, and its overexpression was positively associated with the tumor size and TNM stage of CRC patients. Functionally, UBD significantly accelerated CRC cell viability and proliferation *in vitro* and promoted tumorigenesis *in vivo*. Mechanistically, UBD interacted with p53 in CRC cells, downregulated the expression of p53 by regulating its degradation, shortened the p53 half-life, thereby further affecting the decrease in p21 and the increase in Cyclin D1, Cyclin E, CDK2, CDK4 and CDK6. Moreover, *in vivo* experiments showed that UBD-induced tumor growth in nude mice was dependent on a decrease in p53.

**Conclusions:**

Our study proved that UBD mediates the degradation of p53, thereby facilitating the growth of CRC cells and ultimately promoting the progression of CRC. Therefore, UBD may be a potential therapeutic target and a promising prognostic biomarker for CRC.

## Introduction

Colorectal cancer (CRC) is one of the most common malignant tumors, with increasing morbidity and mortality. Its prevalence is increasing annually, and it seriously threatens human health, with approximately 1.9 million new cases and more than 930,000 new deaths in 2020 worldwide (1). The initiation and progression of CRC is a process of development from adenoma to carcinoma. The activation of oncogenes or the inactivation of tumor suppressor genes in epithelial tissues leads to the hyperplasia of mucosa, resulting in benign adenomas and ultimately cancer (2, 3). Various proteins and signaling pathways are involved in this process. Therefore, it is of great significance to deepen our understanding of the molecular mechanisms which are related to CRC occurrence and development, and provide new perspective for CRC diagnosis and treatment.

Ubiquitination is a kind of posttranslational modification (PTM), that is involved in the regulation of many biological processes, including the cell cycle, differentiation, transcription regulation, signal transmission and damage repair. Ubiquitinated proteins are eventually degraded by the 26S proteasome (4–6). Ubiquitin D (UBD, also called FAT10) is a ubiquitin-like protein, and the N- and C- terminus of UBD are 29% and 36% identical to ubiquitin, respectively. Studies have demonstrated that with the catalysis of an E1 activating enzyme, UBA6, and an E2 conjugating enzyme, UBA6-specific enzyme 1 (USE1), UBD directly binds to its substrate through two glycine residues (GG) in C-terminus to form a UBD-substrate complex, which enters the proteasome system for proteolysis (7–10). Whether this process requires participation of E3 ligase is still unclear (11, 12). UBD can be overexpressed under the stimulation of inflammatory factors (13). Furthermore, researches have shown that UBD is frequently overexpressed in multiple types of cancers (14, 15), and abnormal expression and dysfunction of UBD are closely associated with the occurrence and development of tumors (16). In triple-negative breast cancer (TNBC), UBD is highly upregulated and related to the epirubicin resistance of TNBC patients and may be a potential therapeutic and prognostic indicator in TNBC (17). UBD interacts with WNT1-inducible signaling pathway protein 1 (WISP1), thus inducing its degradation in hepatocellular carcinoma (HCC). On the other hand, UBD can increase the expression of WISP1 mRNA by stabilizing β-catenin, eventually leading to inconsistent expression between WISP1 protein and mRNA, and the degradation of WISP1 protein caused by UBD is dominant and promotes HCC progression (18). A previous study shows that UBD expression is highly upregulated in CRC tissues, and is closely associated with clinical staging and lymph node metastasis of patients with CRC, but not related to tumor size or tumor differentiation (19). In addition, colon cancer patients with high UBD expression have a higher recurrence rate and worse prognosis after 5-FU chemotherapy on the basis of surgery than those with low UBD expression (20, 21). However, the underlying mechanism by which UBD plays a role in the development and prognosis of CRC remains unclear.

Previous researches have confirmed that transcription factor p53 (encoded by the TP53 gene) is a tumor suppressor and play a major role in maintaining genome stability (22). The expression of p53 is upregulated when DNA is damaged, which ultimately inhibits cell cycle progression and induces cell apoptosis (23–25). Mutation, deletion or translocation of p53 usually induces tumorigenesis, which occurs in more than 50% of human tumors (26–28). p53 mutations exist in approximately 40%-50% of sporadic CRC (29). The expression of p53 and its target genes is dysregulated in CRC patients (30, 31). p53 is associated with lymph node positivity, TNM staging and the prognosis of CRC patients (32). Additionally, mass spectrometry analysis has shown that p53 is the interacting protein of UBD (33). However, it remains unclear whether UBD regulates the progression of CRC depending on p53. Here, we demonstrated that UBD downregulates the expression of p53 by regulating its degradation to facilitate the growth of CRC cells.

## Materials and Methods

### Reagents and Antibodies

The lentivectors pGreenPuro (SI505A-1) and pCDH-CMV-MCS-EF1α-GreenPuro (CD513B-1) were purchased from System Biosciences (SBI, Palo Alto, CA, USA). Lentivira packaging plasmid psPAX2 (#12260) and envelope plasmid pMD2.G (#12259) were gifts from Didier Trono (Addgene, Cambridge, MA, USA). Cycloheximide (CHX) and MG132 were obtained from Selleck Chemicals (Houston, TX, USA). Details of antibodies used in this study are shown in **Supplementary Table 1**.

### Human Tissue Samples

In total, 40 pairs of CRC tissue samples and corresponding adjacent nontumor tissue samples were obtained from patients who underwent CRC resection at The First Affiliated Hospital of Chongqing Medical University (Chongqing, China). The case screening criteria were as follows: pathological examination confirmed that the excised sample was CRC, patients who received radiotherapy and/or chemotherapy before surgery and hereditary CRC, such as Lynch syndrome were excluded. All participants in this study provided written informed consent, and this study was approved by the Ethics Committee of The First Affiliated Hospital of Chongqing Medical University.

### Cell Lines and Cell Culture Conditions

Seven human CRC cell lines including HCT116, SW480, SW620, Caco-2, HT-29, LoVo and RKO, and HEK293T (human embryonic kidney cell line 293T) were purchased from the Cell Bank of Chinese Academy of Science (Shanghai, China). All cell lines were cultivated in RPMI-1640 or DMEM (Gibco, Grand Island, NY, USA) containing 10% fetal bovine serum (FBS, NATOCOR, Cordoba, Argentina) and 1% penicillin-streptomycin solution (HyClone, Logan, UT, USA) in a humidified environment at 37°C with 5% CO_2_.

### Immunohistochemical (IHC) Staining

Human colorectal cancer tissues and specimens taken from nude mice were fixed with 10% paraformaldehyde, embedded in paraffin, and cut into 4 μm slices. The paraffin sections were baked at 55°C for 4 hours, and then soaked in fresh xylene for 20 minutes to dewax. Sections were hydrated with alcohol at different concentrations and deionized water, and then boiled in citrate buffer solution (10 mmol/L pH 6.0) at 100°C for 20 minutes to repair the antigen. Subsequently, these sections were incubated with 0.05% Triton X-100 (Dingguo, Beijing, China) at room temperature for 30 minutes. A rabbit streptavidin-biotin detection kit (SP-9001, ZSGB, Beijing, China) was adopted for staining the sections. Briefly, sections were treated with endogenous peroxidase blocker for 10 minutes and blocked with goat serum for 30 minutes at room temperature, and then incubated overnight at 4°C with primary antibodies against UBD (1:100, DF7373), p53 (1:200, 21891-1-AP), or Ki67 (1:500, ab92742). Afterwards, sections were incubated with biotin-conjugated goat anti-rabbit IgG for 15 minutes and HRP-labeled streptavidin working solution for 15 minutes at room temperature. Finally, the signals were visualized by employing brown 3,3’-diaminobenzidine tetrahydrochloride (DAB) staining (ZLI-9018, ZSGB, Beijing, China) and then counterstaining with hematoxylin blue. Tissue samples incubated with PBS instead of primary antibody were used as a negative control. Whole histological sections were digitized using the Pannoramic DESK scanner (3DHISTECH, Budapest, Hungary), and images were acquired at 200× or 400× magnification using the CaseViewer 2.4 software module (3DHISTECH, Budapest, Hungary). The staining score was assessed as previous described (34).

### RNA Extraction, cDNA Synthesis and Quantitative Real-Time Polymerase Chain Reaction (qRT-PCR)

TRIzol reagent (Invitrogen, Carlsbad, CA, USA) was used for total RNA isolation from CRC cells and tissues. cDNA was generated with the Prime-Script™ RT reagent Kit (Takara, Tokyo, Japan) according to the manufacturer’s instructions. qPCR was performed with 2x SYBR Green qPCR Master Mix (Bimake, Houston, TX, USA). Each cDNA sample was subjected to PCR amplification reactions in triplicate. Relative RNA levels were calculated using the comparative CT (2^-ΔΔCT^) method. Glyceraldehyde 3-phosphate dehydrogenase (GAPDH) was employed as a loading control. The primers for PCR are listed in **Supplementary Table 2** and were provided by TsingKe Company (Chongqing, China).

### Protein Extraction, Western Blotting and Immunoprecipitation

RIPA lysis buffer (Beyotime, Shanghai, China) supplemented with 1% phenylmethylsulfonyl fluoride (PMSF, Dingguo, Beijing, China) was used for protein extraction of cells and tissues. The protein concentration was measured with a BCA kit (Dingguo, Beijing, China) according to the manufacturer’s instructions. Protein samples (40 μg) were separated by 10% sodium dodecyl sulfate polyacrylamide gel electrophoresis (SDS-PAGE) and then transferred to a polyvinylidene difluoride (PVDF) membrane. The membrane was blocked with TBST containing 5% skimmed milk at room temperature for 1.5 hours and incubated with specific primary antibody diluted with 5% BSA at 4°C overnight. After incubation with secondary antibody for 2 hours at room temperature, the protein signals were detected using a ECL chemiluminescence kit (Advansta, San Jose, CA, USA). Immunoprecipitation was performed as previously described (34). Grayscale value analysis of all protein bands was performed *via* ImageJ 1.46r software (NIH, Bethesda, MD, USA).

### Plasmid Construction, Transfection and Establishment of Stable Cell Lines

Short hairpin RNA (shRNA) directed against human UBD, p53, UBA6, USE1 or the negative control was generated by annealing two complementary shRNA oligonucleotide strands synthesized by TsingKe Company and then inserting them into the pGreenPuro vector. The shRNA target sequences are listed in **Supplementary Table 3**. For overexpression of UBD, the cDNA sequence containing the UBD open reading frame (ORF) was synthesized by TsingKe Company and cloned into the EcoRI/BamHI sites of the vector pCDH-CMV-MCS-EF1α-GreenPuro. HEK293T cells were cotransfected with lentiviral vectors (or control lentivirus vectors), auxiliary packaging plasmid psPAX2 and pMD2.G using Lipofectamine 2000 transfection reagent (Invitrogen, Carlsbad, CA, USA) following the manufacturer’s protocol. The lentivirus supernatant was collected after 48 hours. CRC cells were infected with lentiviral supernatant supplemented 5 μg/mL polybrene (Beyotime, Shanghai, China), and stably transfected cell lines were screened using 1 μg/mL puromycin (Beyotime, Shanghai, China) for 2 to 3 weeks.

### Cell Counting Kit-8 (CCK-8) Assays

A CCK-8 kit (MCE, Monmouth Junction, NJ, USA) was used to detect the viability of CRC cells. HCT116 and RKO cells at logarithmic growth phase (1 × 10^3^) were seeded in 96-well plates in triplicate. Once the cells adhered, CCK-8 reagents (10 μL/well) were added at the indicated times and incubated for 2 hours. Then the optical density (OD) of each well was measured at 450 nm using a spectrophotometric plate reader. The measurements were performed once per day for 5 continuous days.

### Plate Colony Formation Assay

Control and treated CRC cells were seeded at a density of 2 × 10^3^ cells/well in 6-well plates. After culturing in complete medium for 12-14 days, cells were fixed with 4% paraformaldehyde for 30 minutes, and stained with 0.1% crystal violet for 15 minutes. Then the numbers of colonies containing > 50 cells were counted *via* ImageJ 1.46r software.

### EdU Assays

Cell proliferation was measured using a 5-ethynyl-2’-deoxyuridine (EdU) Cell Proliferation Assay Kit (C0075S, Beyotime, Shanghai, China) as described in the manufacturer’s instructions. CRC cells were cultured at a density of 3 × 10^5^ cells/well in a 12-well plate. After attachment, the cells were exposed to EdU labeling medium for approximately 2 hours at 37°C. Afterwards, the cells were fixed with 4% formaldehyde for 15 minutes and treated with enhanced immunostaining permeabilization buffer (P0097, Beyotime, Shanghai, China) for 15 minutes at room temperature for permeabilization, after which the click reaction mixture were added to the cells and incubated for 30 minutes. Next, the nuclei of the cells were stained with Hoechst 33342 in the dark at room temperature for 10 minutes and visualized under a confocal laser scanning microscope (ZEISS, Jena, Germany). Experiments were performed in triplicate.

### Flow Cytometry

Cell cycle and apoptosis analyses were performed using flow cytometry by the Institute of Life Sciences, Chongqing Medical University (Chongqing, China). For cell cycle analysis, 1×10^6^ cells were harvested by treatment with 0.25% trypsin and fixed with prechilled 75% ethanol at 4°C overnight.

### Protein Half-Life Detection

HCT116 and RKO cells were separately transfected with shNC, shUBD#1, pCDH or pCDH-UBD plasmids and treated with 100 μg/mL cycloheximide (CHX). after culturing for 0, 2, 4 and 8 hours, the treated cells were collected for protein extraction and Western blotting. A total of 40 μg protein was loaded and anti-UBD or anti-p53 was used as the primary antibody. Relative expression of p53 (normalized to GAPDH protein expression) was evaluated using ImageJ software and the half-life of p53 was calculated.

### Tumorigenicity Assay *In Vivo*

5-6 week-old male BALB/c nude mice were purchased from SPF Biotechnology Co., Ltd. (Beijing, China). All animals were housed under specific pathogen-free (SPF) conditions at the Animal Experimental Laboratory Center of Chongqing Medical University. For *in-vivo* tumorigenicity assays, 1 × 10^7^ UBD stable knockdown or overexpression HCT116 cells (suspended in 100 μL of PBS) were injected subcutaneously into the right flanks of mice (5 mice/group). The survival of the mice and tumor growth status were monitored. Tumor volume was measured every 4 days and calculated as follows: volume (mm^3^)= 0.5 × (largest diameter) × (smallest diameter)^2^. Mice were euthanized 24 days after inoculation, and the tumors were harvested, photographed, and weighed. Animal care and handling procedures were approved by the Ethics Committee of Animal Experiments of Chongqing Medical University.

### Statistical Analysis

GraphPad Prism 8 software (GraphPad Software, San Diego, CA, USA) was employed for statistical analysis. The data were expressed as the means ± standard deviation (SD) from three independent experiments. Pearson’s chi-square test was used to measure the relationship between UBD expression and other enumeration data of clinical characteristics in CRC patients. Student’s t test (unpaired, two-tailed) was used to analyse the differences between two groups. The correlation of UBD and p53 was investigated using the Spearman correlation test. The results were considered statistically significant at *p*-value < 0.05, and significance was indicated as follows: *p < 0.05, **p < 0.01 and ***p < 0.001.

## Results

### UBD Is Significantly Upregulated and Correlates With Clinical Parameters in CRC

**Figure 1:**
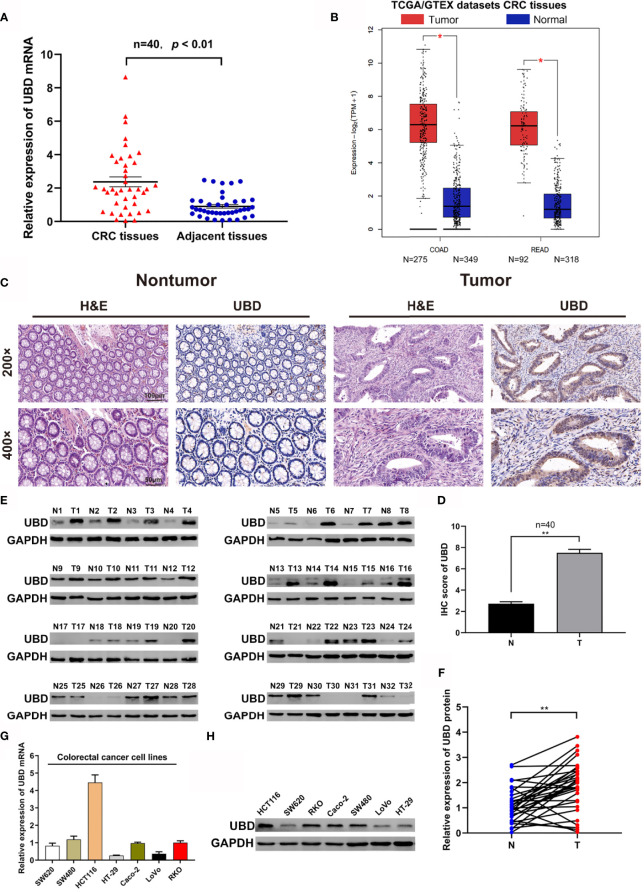
The expression of UBD in CRC. **(A, B)** The expression level of UBD was detected in 40 cases of CRC tissue samples **(A)** and the TCGA/GTEX datasets **(B)**. **(C)** H&E staining was used to detect the benign and malignant tissues, IHC staining of UBD was used to determine the expression of UBD protein in CRC tumor tissues and adjacent nontumor tissues (magnifications, 200× and 400×; scale bar, 100 μm and 50 μm). **(D)** IHC score of UBD in CRC tumor tissues (T) and adjacent nontumor tissues (N) (n = 40, **p < 0.01). **(E)** Western blot analysis of UBD protein expression from CRC tissues, GAPDH was used as a loading control. **(F)** Relative expression of UBD protein was quantized by analyzing grayscale value of Western blotting bands in 32 pairs of CRC tissues. (n=32, **p < 0.01). **(G, H)** Relative mRNA level **(G)** and protein level **(H)** of UBD in seven CRC cell lines including SW620, SW480, HCT116, HT-29, Caco-2, LoVo and RKO. GAPDH was used as a loading control. CRC, colorectal cancer; COAD, Colon adenocarcinoma; READ, Rectal adenocarcinoma; H&E, hematoxylin and eosin; IHC, immunohistochemistry; UBD, Ubiquitin D; GAPDH, glyceraldehyde 3‐phosphate dehydrogenase.

To investigate the expression of UBD in CRC, the mRNA level was detected in 40 pairs of CRC tumor and nontumor tissues by qRT-PCR. The expression of UBD was clearly upregulated in tumor tissues compared with nontumor tissues (**Figure 1A**). In addition, the expression of UBD was significantly higher in 367 CRC tumor tissues than in 667 normal tissues from the TCGA/GTEX datasets with an online platform (http://gepia2.cancer-pku.cn) (35) (**Figure 1B**). Next, IHC staining was used to assess the protein expression and localization of UBD in 40 CRC samples. The results showed that UBD expression was clearly increased and was mainly located in the nucleus of cancer cells (**Figures 1C, D**). Western blotting also revealed that the protein expression level of UBD was higher in CRC tumors than in adjacent nontumor tissues (**Figures 1E, F**). Moreover, UBD expression in SW620, SW480, HCT116, HT-29, Caco-2, LoVo, and RKO cell lines was detected using qRT-PCR and Western blotting (**Figures 1G, H**). Western blotting results showed that HCT116, RKO, Caco-2 and SW480 have relatively high UBD expression, and HCT116 and RKO cells were used to perform further experiments.

Next, we investigated the associations between UBD expression levels and clinicopathological features in CRC patients, the patients were divided into two groups (UBD high and low expression) as previous described (34). We found that UBD expression was significantly associated with tumor size (*p* = 0.037) and TNM stage (*p* = 0.017) but not with patient sex, age or tumor location (**Table 1**). Taken together, we concluded that UBD is frequently upregulated in human CRC and that UBD has a potential role as a tumor promoter in CRC.

**Table 1:** Correlation between UBD expression and clinicopathologic characteristics in CRC patients.

Parameters	Total case	UBD		P-value
		High (%)	Low (%)	
	40	23 (57.5)	17 (42.5)	
Gender				0.676
Men	22	12 (54.5)	10 (45.5)	
Women	18	11 (61.1)	7 (38.9)	
Age(Years)				0.433
> 55	24	15 (62.5)	9 (37.5)	
≤ 55	16	8 (50)	8 (50)	
Location				0.554
Colon	19	10 (52.6)	9 (47.4)	
Rectal	21	13 (61.9)	8 (38.1)	
Metastasis				0.525
Lymph nodes	16	9 (56.3)	7 (43.7)	
Distant	5	2 (40)	3(60)	
Stages				0.017*
I-II	15	5 (33.3)	10 (66.7)	
III-IV	25	18 (72)	7 (28)	
Tumor size				0.037*
> 40mm	17	13 (76.5)	4 (23.5)	
≤ 40mm	23	10 (43.6)	13 (56.5)	

*p < 0.05.

### UBD Positively Promotes the Proliferation of CRC Cells and Cell Cycle Progression *In Vitro*

**Figure 2:**
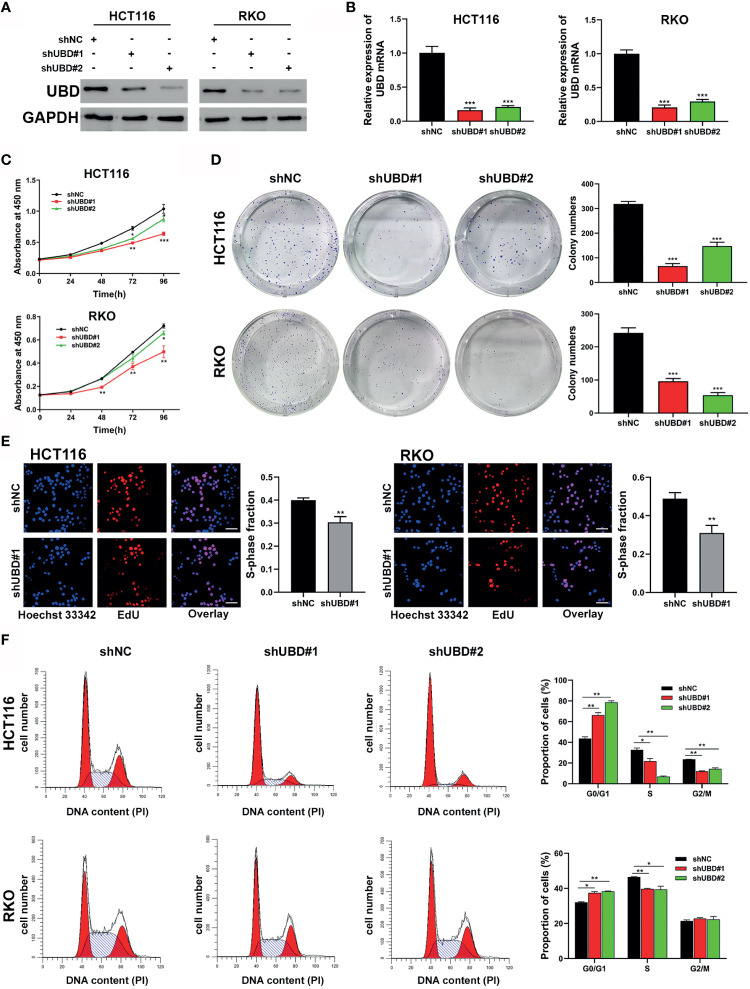
Knockdown of UBD inhibits the CRC cell growth and cycle progression *in vitro*. **(A, B)** Knockdown efficiency of UBD in HCT116 and RKO cells detected using Western blotting **(A)** and qRT-PCR **(B)** (***p < 0.001). GAPDH was used as an endogenous control. **(C)** CCK-8 assay was used to analyze the cell viability of UBD stable knocked down HCT116 and RKO cells (*p < 0.05; **p < 0.01; ***p < 0.001). **(D, E)** The effect of UBD downregulating on the proliferation of HCT116 and RKO cells compared with negative control were analyzed by colony formation assays **(D)** and EdU assay **(E)**, magnification, 400×; scale bar, 100 μm). Quantitative results of colony numbers and EdU positive cells are presented in data graphs (**p < 0.01; ***p < 0.001). **(F)** Flow cytometry assays were performed to analyze cell cycle in HCT116 and RKO UBD stable knockdown cell lines (*p < 0.05; **p < 0.01). shNC, short hairpin negative control; CCK-8, Cell Counting Kit-8; EdU, 5-ethynyl-2’-deoxyuridine.

As high UBD protein expression was significantly associated with tumor size, we speculated that UBD may function in CRC proliferation. To explore the relationship between UBD and CRC cell growth, shRNAs shUBD#1 and shUBD#2 were designed to mediate UBD knockdown. HCT116 and RKO cells were transfected with shUBD#1, shUBD#2 or empty control vector shNC separately, and qRT-PCR and Western blotting were generated to evaluate the efficiency of shUBD#1 and 2. The results showed that shUBD#1 and 2 successfully knocked down UBD protein (**Figure 2A**) and mRNA expression (**Figure 2B**).

**Figure 3:**
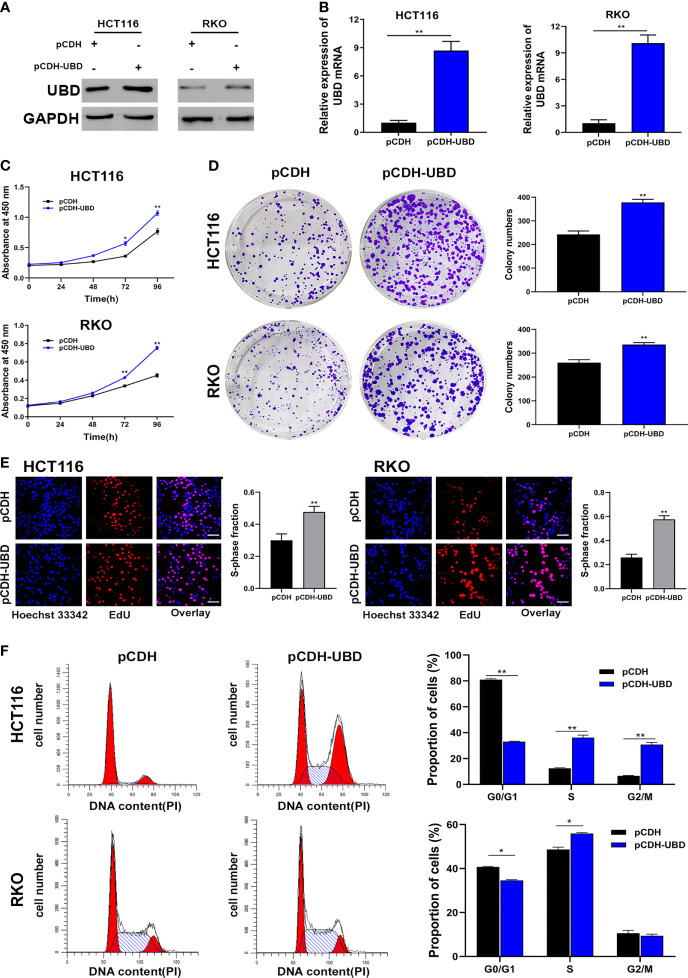
Upregulated expression of UBD promotes the CRC cell proliferation and cycle progression *in vitro*. **(A, B)** Western blot **(A)** and qRT-PCR **(B)** analyses of UBD protein and mRNA levels in CRC cells after UBD overexpression (**p < 0.01). **(C)** CCK-8 assay was used to analyze the cell viability of UBD stable overexpressed HCT116 and RKO cells (*p < 0.05; **p < 0.01). **(D, E)** The effect of UBD overexpression on the growth of HCT116 and RKO cells compared with negative control were analyzed by colony formation assays **(D)** and EdU assay **(E)**, magnification, 400×; scale bar, 100 μm). Quantitative results of colony numbers and EdU positive cells are presented in data graphs (**p < 0.01). **(F)** Flow cytometry assays were performed to analyze cell cycle in HCT116 and RKO UBD stable overexpression cell lines (*p < 0.05; **p < 0.01).

Then, UBD function in CRC cells were analyzed. First, a CCK-8 assay was performed to detect the viability of the transfected cells. The data showed that the growth dynamics of HCT116 and RKO cells significantly decreased when transfected with shUBD (**Figure 2C**). Next, cell colony formation assays and EdU assays were conducted to further examine the effect of UBD on cell proliferation. We found that UBD knockdown suppressed the proliferation ability of CRC cells (**Figures 2D, E**). Additionally, flow cytometry showed that UBD knockdown markedly blocked the cell cycle at the G1 phase in HCT116 and RKO cells (**Figure 2F**). No significant changes in apoptosis were observed in HCT116 and RKO cells when UBD was knocked down compared to control cells (**Supplementary Figure 1**). Then, we constructed the UBD overexpression vector, pCDH-UBD. HCT116 and RKO cells were transfected with pCDH-UBD or the control vector pCDH. UBD expression level was detected in the transfected cells using Western blotting and qRT-PCR, and the results showed that UBD was successfully overexpressed (**Figures 3A, B**). After UBD was overexpressed, both the viability and proliferation ability of HCT116 and RKO cells were increased compared with those of the scramble group (**Figures 3C–E**). G0/G1 to S cell cycle progression was also promoted (**Figure 3F**). Taken together, these results demonstrated that UBD can promote CRC cell proliferation and cell cycle G0/G1 to S progression *in vitro.*

### UBD Negatively Regulates p53 Expression in CRC

The above results demonstrated that UBD is associated with CRC cell growth *in vitro*. We next examined the molecular mechanisms underlying UBD-regulated cell growth. A previous mass spectrometry screen identified p53 as a UBD-interacting protein (33). Based on this, we speculated that there might be a functional association between UBD and p53.

**Figure 4:**
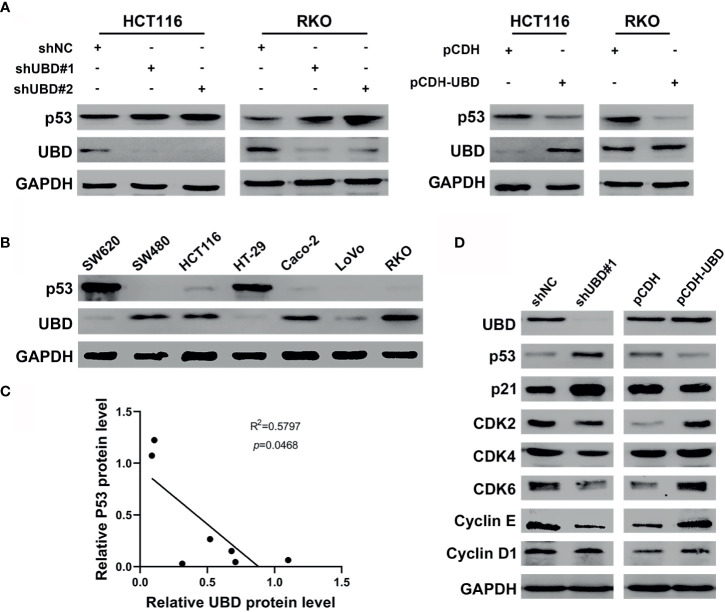
UBD negatively regulates the expression levels of p53 in CRC. **(A)** The protein expression level of p53 in HCT116 and RKO cells transfected with stable knockdown or overexpression plasmid. **(B)** Expression level of UBD and p53 protein were detected in seven CRC cell lines. **(C)** Pearson correlation analyzed the correlation between UBD and p53 protein expression level in CRC cells (R^2^ = 0.5797; *p* = 0.0468). **(D)** Western blotting was used to detected the protein expression of downstream genes of p53 pathway in HCT116 cells transfected with stable knockdown or overexpression plasmid. GAPDH was used as an endogenous control. p53: tumor suppressor p53.

To illuminate whether p53 is a target protein of UBD in CRC cells, the change of p53 protein expression level along with UBD in HCT116 and RKO cells were assessed using Western blot analyses. The results showed that UBD knockdown significantly increased p53 protein expression, whereas overexpression of UBD reduced the expression of p53 protein (**Figure 4A**) in HCT116 and RKO cells. To further study the relationship between UBD and p53, we detected the protein levels of UBD and p53 in seven CRC cell lines using immunoblotting. In the seven CRC cell lines, there was a negative correlation between the protein expression levels of p53 and UBD (**Figures 4B, C**). Additionally, immunoblot analysis in HCT116 cells revealed that the knockdown of UBD led to increases in p21 and decreases in Cyclin D1, Cyclin E, cyclin-dependent kinase 2 (CDK2), CDK4 and CDK6 expression (**Figure 4D**), which are proteins related to cell cycle regulation. In contrast, overexpression of UBD generated the opposite expression trend of these cell cycle-related proteins (**Figure 4D**). These results indicated that the p53 expression might be downregulated by UBD in CRC cells to promote cell cycle progression and cell growth.

### p53 Is Required for UBD to Regulate the Growth of CRC Cells

**Figure 5:**
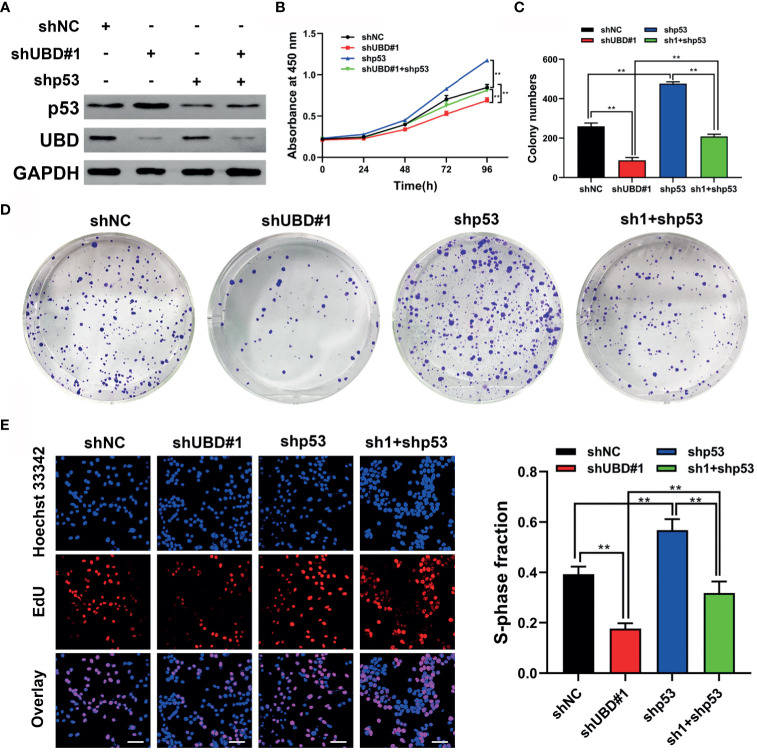
p53 is required for UBD to regulate growth of CRC cells. **(A)** Western blot analysis of UBD and p53 protein expression in HCT116 cells after UBD knockdown with or without p53 knockdown. **(B)** Cell viability of shp53 and shUBD#1 co-transfection in HCT116 cells evaluated by CCK-8 assay (**p < 0.01). **(C-E)** Results of colony formation assay **(C, D)** and EdU assays **(E)** showing the proliferation of CRC cells stably transfected with shUBD#1 in the presence or absence of shp53 **(E)**, magnification, 400×; scale bar, 100 μm). Quantitative results of colony numbers and EdU positive cells are presented in data graphs (**p < 0.01). sh1, shUBD#1.

As mentioned above, p53 has been identified as a target protein of UBD, and we hypothesized that UBD might elevate CRC cell proliferation by inhibiting p53 expression. Therefore, we constructed a short-hairpin RNA targeting p53 (shp53), cotransfection HCT116 cells with shUBD#1 plasmid, and verified the protein expression of p53 and UBD by Western blotting (**Figure 5A**). CCK-8 assays showed that knockdown of UBD significantly depleted CRC cell viability, which could be reversed by reduction of p53 (**Figure 5B**). Similarly, colony formation and EdU experiments indicated that knockdown of UBD markedly impaired CRC cell growth. Furthermore, a decrease in p53 promoted the proliferation of CRC cells which could block the inhibition of CRC cell proliferation when UBD was knocked down (**Figures 5C–E**). Taken together, these data suggested that p53 is indispensable for the proliferative function of UBD in CRC cells.

### UBD Inhibits p53 Stability by Enhancing the Proteolysis of p53

**Figure 6:**
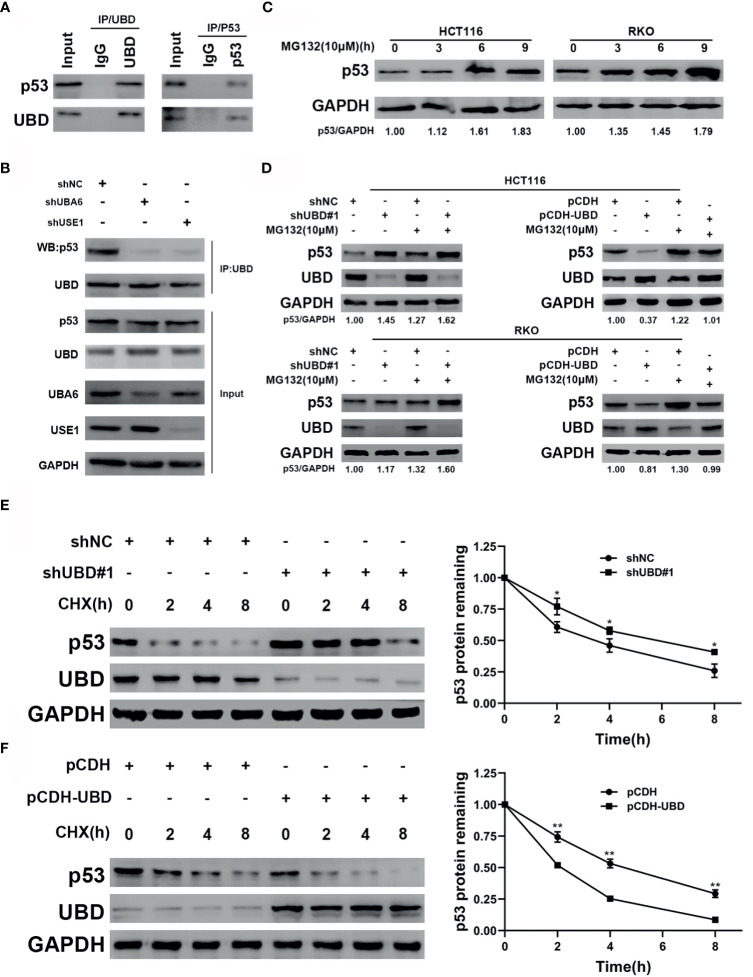
UBD inhibits p53 stability by enhancing the protein degradation of p53. **(A)** Immunoprecipitaion of endogenous UBD and p53 in HCT116 cells. **(B)** UBD-p53 complexes were examined in HCT116 cells transfected with the indicated shRNAs **(C)** Western blot analysis of p53 expression in cells treated with MG132 (10 μM) for the indicated times. **(D)** After transfection with the shNC, shUBD#1, pCDH or pCDH-UBD plasmids, p53 expression in HCT116 and RKO cells was observed upon addition of MG132 (10μM) for 6 hours by using Western blotting. **(E**, **F)** HCT116 cells were transfected with shNC or shUBD#1 **(E)**, RKO cells were transfected with pCDH or pCDH-UBD **(F)**. Then treated with 100 μg/mL CHX for the indicated times. Western blotting was used to detect the UBD and p53 expressions. Quantitative analysis was conducted on p53 level in Western blotting, the half-life time of p53 was calculated (*p < 0.05; **p < 0.01). GAPDH was used as an endogenous control. CHX, cycloheximide.

To verify the mechanism by which UBD regulates the expression of p53, immunoprecipitation was performed to detect whether UBD interacted with p53 directly. The results showed that UBD and p53 can bind to each other in HCT116 cells (**Figure 6A**). Previous studies confirmed that, under the catalysis of E1 (UBA6) and E2 (USE1), UBD is able to bind to substrates to form UBD-substrate complexes and lead to proteasome degradation of the substrate (8, 18, 36). To test the changes in UBD-p53 complex formation when the expression of E1 and E2 enzymes were reduced in HCT116 cells, UBA6 and USE1 were knocked down respectively. Results showed that downregulation of UBA6 or USE1 obviously inhibited the formation of the UBD-p53 complex (**Figure 6B**). Taken together, these results revealed that UBD interacts with p53 and p53 can be modified by UBD/FAT10ylation.

In addition, we supposed that UBD might target p53 protein degradation through the proteasome system. To prove this hypothesis, we treated HCT116 and RKO CRC cells with MG132, a proteasome inhibitor, for the indicated concentrations and times, and the results showed that the protein expression of endogenous p53 significantly accumulated over time following MG132 treatment (**Figure 6C**). To further clarify the effect of UBD on the regulation of p53 protein degradation, we transfected shNC, shUBD#1, pCDH, and pCDH-UBD expression vectors into HCT116 and RKO cells. After incubation with or without MG132 at specific times, Western blot analysis was used to test the expression of UBD and p53. The results demonstrated that the protein level of p53 was upregulated when UBD was knocked down, and the increase in UBD expression by pCDH-UBD largely decreased endogenous p53 protein expression. In addition, the expression of p53 in the MG132 treatment group was higher than that in the group without MG132 treatment (**Figure 6D**), suggesting that UBD can negatively regulate p53 expression in the proteasome degradation pathway. Moreover, to analyze the dynamics of p53 degradation, UBD stably expressed HCT116 and RKO cells were treated with cycloheximide (CHX), an inhibitor of protein synthesis. The results showed that, compared to HCT116-shNC, the half-life of p53 in HCT116-shUBD#1 cells was increased from 4 hours to approximately 6 hours (**Figure 6E**). Conversely, overexpression of UBD in RKO cells significantly reduced the half-life of p53 compared with that in the control cells (**Figure 6F**). These results demonstrated that UBD inhibits p53 stability by facilitating the protein degradation of p53. E3 ubiquitin ligase murine double minute (MDM2) is recognized as one of the most critical negative regulator for p53 protein stability. MDM2 binds to the transactivation domain of p53 and blocks this area, thus inhibiting p53-mediated transcriptional activation and inducing the ubiquitination of p53, finally leading to its degradation (37, 38). We assumed that the effect of UBD on p53 related to MDM2 and tested the effect of UBD on the expression of MDM2, however, the expression of MDM2 did not change significantly (**Supplementary Figure 2**).

### UBD Accelerates CRC Cell Growth by Downregulating the Expression of p53 *In Vivo*

**Figure 7:**
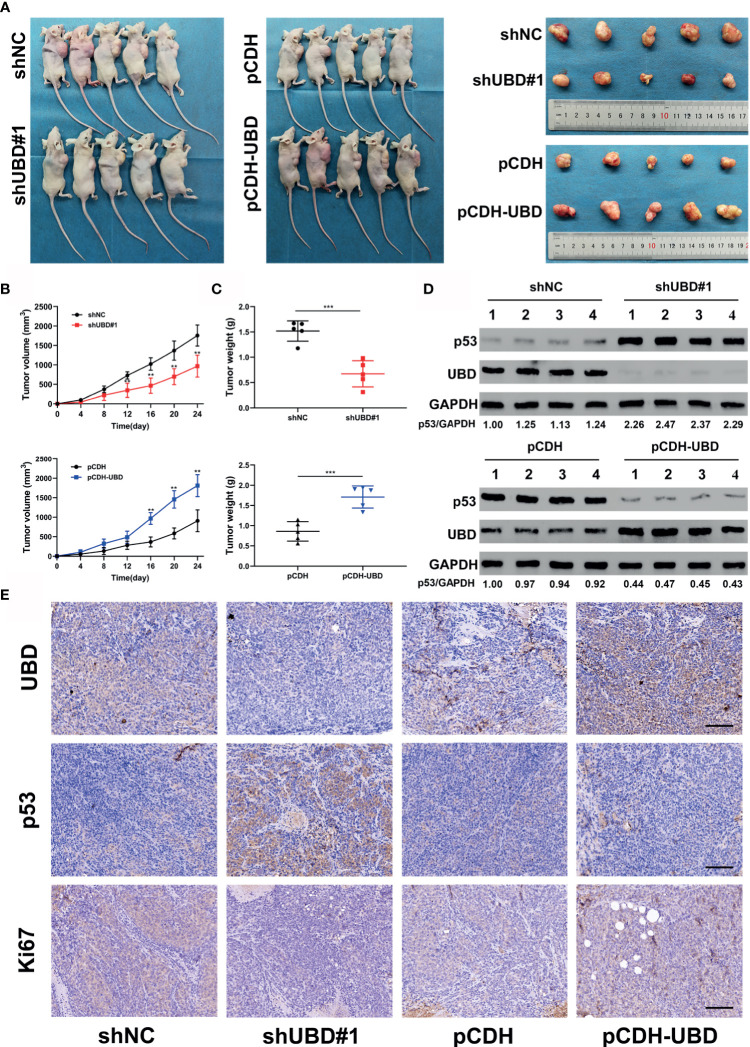
UBD accelerates CRC cell growth by downregulating the expression of p53 *in vivo.* **(A–C)** HCT116 cells that stablely express shNC, shUBD#1, pCDH or pCDH-UBD were subcutaneously injected into the right armpits of nude mice (five mice per group). After 24 days of growth, the mice were euthanized, and the tumors were harvested, photographed **(A)**; tumor volumes were measured periodically after injection and tumor growth curves were drawn **(B)**; tumor weights were measured **(C)** (**p < 0.01; ***p < 0.001). **(D)** Protein levels of UBD and p53 in tumors from HCT116 injected cells in which UBD was stably downregulated or overexpressed (n=4 per group). GAPDH was used as an loading control. **(E)** IHC staining of UBD, p53 and Ki67 in xenograft tumor tissues (scale bar, 100 μm).

The above data confirmed that UBD promotes the proliferation of CRC cells *in vitro* by regulating the expression of p53. To further verify whether UBD plays an oncogenic role *in vivo*, HCT116 cells that stably express shNC, shUBD#1, pCDH or pCDH-UBD were subcutaneously injected into the right armpits of nude mice (five mice per group). The volume of tumors was measured every 4 days, continually for 24 days. And then the mice were euthanized, and the tumors were harvested, photographed, and weighed (**Figure 7A**). We found that the tumors of mice in the HCT116-shUBD#1 group were smaller and lighter than those of the shNC mice. However, a marked increase in tumor size and weight could be seen in the UBD overexpression group compared with the control group (**Figures 7B, C**). These results indicated that UBD promotes CRC tumorigenesis *in vivo*. To further confirm the effect of p53 on UBD-mediated tumor growth *in vivo*, immunoblots for UBD and p53 were adopted in these tumor tissues. The results showed that knockdown of UBD promoted p53 expression, whereas UBD overexpression had opposite effects (**Figure 7D**). Consistent with the Western blot analysis, IHC assays of the isolated tumors also confirmed these results (**Figure 7E**, **Supplementary Figure 3**), and Ki-67 staining revealed increased cell proliferation in HCT116 xenografts overexpressing UBD. Conversely, cell proliferation was inhibited when UBD was silenced (**Figure 7E**). Taken together, these findings suggested that UBD promotes CRC proliferation by downregulating the expression of p53 *in vivo*.

## Discussion

CRC has undergone a process of development from adenoma to carcinoma, which involves a variety of proteins and signaling pathways (3). Although the diagnosis and treatment of CRC have made great progress in recent years, the 5-year survival rate of CRC is still poor, especially for patients with advanced CRC (39). This is mainly due to the insufficient understanding of the molecular mechanism of its occurrence and development.

UBD is a ubiquitin-like protein. And its function of targeting protein degradation is similar to that of ubiquitin, and it is currently the only known ubiquitin-like protein that can directly mediate ubiquitin-independent proteasome degradation (40). Evidence has confirmed that UBD is overexpressed in a variety of tumors, including CRC, and is related to tumor proliferation, metastasis and prognosis (14, 41, 42). The results from the TCGA/GTEX datasets and our clinicopathological specimens also confirmed that the expression level of UBD in CRC tumor tissues was significantly higher than that in nontumor tissues. Clinical features demonstrated that overexpressed UBD was positively associated with tumor size and TNM stage (**Table 1**), which was consistent with previous studies (19–21). Furthermore, we investigated the biological function of UBD in CRC both *in vitro* and *in vivo*. We found that knocking down UBD significantly inhibited the viability and capacity of colorectal cancer cells to grow *in vitro*, arresting the cells in G1 phase. *In vivo* experiments also confirmed that the growth of colorectal tumors was suppressed when UBD was knocked down. In contrast, stable overexpression of UBD had the opposite effect. Taken together, these results suggested that UBD may serve as an oncogene in CRC.

The tumor suppressor p53 plays an essential role in promoting the development of CRC from adenoma to carcinoma. The amount of p53 protein keeps low in normal cells, and the expression level of p53 is mainly determined by ubiquitin-dependent proteasome degradation (43). When activated, p53 upregulates the transcription of MDM2, and MDM2 binds to and ubiquitinates it for proteasomal degradation (37). The interaction between p53 and MDM2 is a negative feedback loop, and MDM2 acts as a negative regulator of p53 level and activity (44, 45), which has become a promising therapeutic strategy in cancer treatment (26, 28). A previous study had proved that both p53 and MDM2 are interacting proteins of UBD (33). In this study, we confirmed that the expression level of p53 protein was opposite to these of UBD both *in vitro* and *in vivo*. Additionally, we found that UBD interacted with p53, leads to a decrease in p53 protein expression by UBD/FAT10ylation-mediated degradation and shorten the half-life of p53, suggesting that UBD might promote tumorigenesis of CRC by inhibiting the function of p53. However, the expression of MDM2 did not change significantly and we speculated that UBD regulates the expression of p53 may not due to the interaction of p53 and MDM2. When exposed to cellular stress, the p53 signaling pathway was activated to regulate the cell cycle. Cyclins and cyclin-dependent kinases (CDKs) are key regulators that control cell cycle procession. p21 is a member of the CDK inhibitor family and a downstream target gene of p53 that stimulates arrest of the cell cycle at G1 to S phase and G2 to M phase (29). Our results showed that UBD regulates p53 downstream molecules, including p21, CDKs and Cyclins. To further explore whether UBD-mediated CRC growth depends on p53, loss-of-function experiment was performed and showed that p53 could repair the functional inhibition caused by UBD knockdown.

According to previous studies, p53 is covalently modified by UBD overexpression in HEK293 cells, leading to an enhancement of p53 transcriptional activity with abnormal conformation (46). However, upon stimulation by inflammatory factors, UBD is overexpressed, and p53 transcriptional activity is decreased; conversely, silencing of UBD causes the opposite effect (47). In addition, p53 binds to an inhibitory site of the UBD promoter to negatively regulate the promoter activity and mRNA expression of UBD, and endogenous UBD gene expression is increased when endogenous p53 is silenced in HEK293 cells, indicating that UBD might be a downstream gene of the p53 pathway (48). There seems to be a negative feedback regulation underlying the mechanism of UBD and p53. A study also confirmed that the mRNA and protein expression of UBD are positively correlated with that of mutant p53 in gastric cancer tissues, both of which are overexpressed and closely associated with lymph node metastasis, advanced TNM stage and a terrible prognosis (49). There seems to be a complicated mechanism for the mutual regulation of UBD and p53, and further investigation is needed.

**Figure 8:**
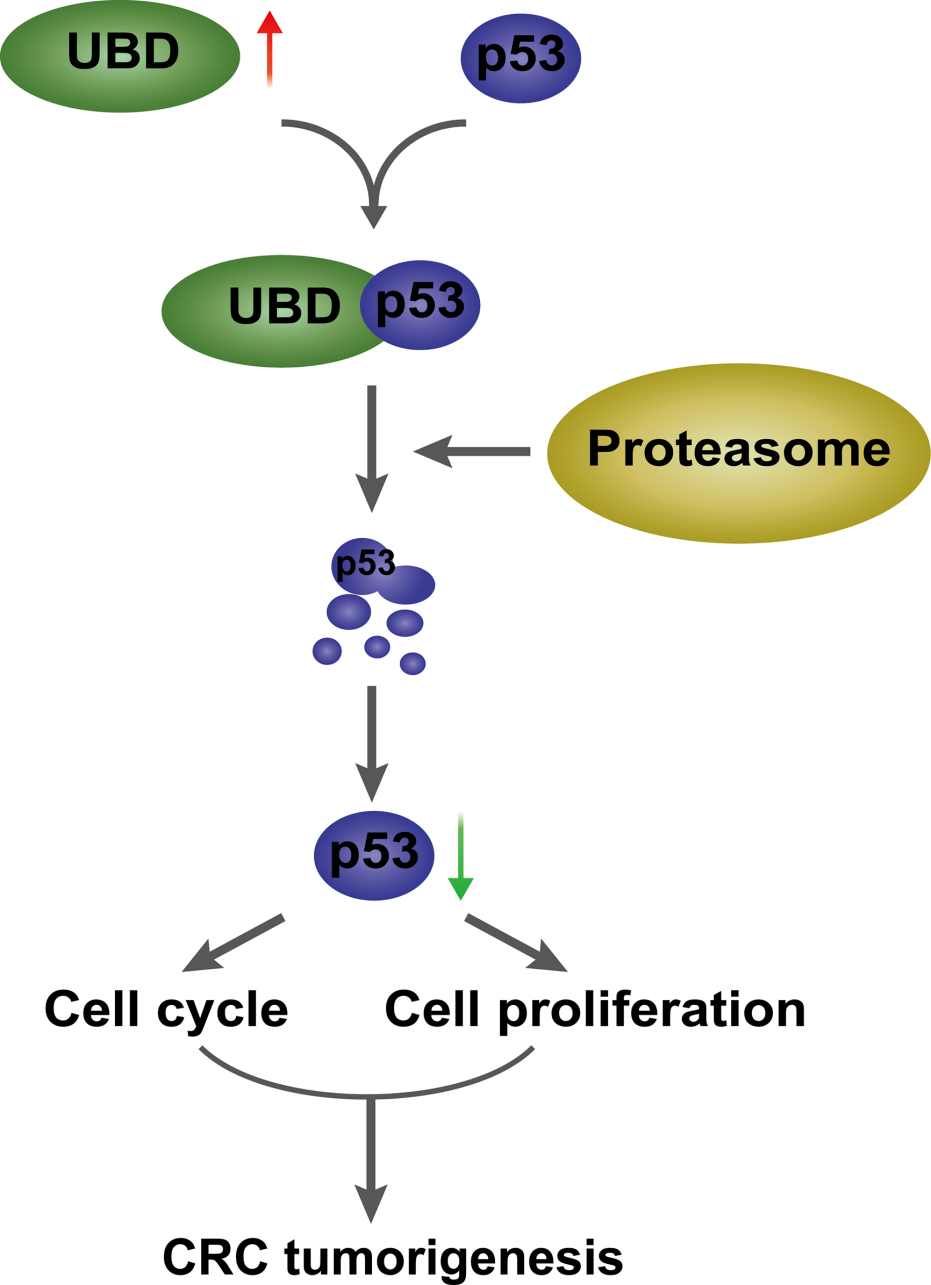
A proposed model of UBD/p53 promoting CRC cell proliferation and tumorigenesis. UBD is upregulated in CRC, and may interact with p53 and facilitate p53 degradation through the proteasome pathway, which further promotes cell proliferation and cell cycle progression, ultimately, promotes CRC tumorigenesis.

In conclusion, UBD is frequently overexpressed in CRC and closely associated with the clinicopathological features of CRC patients. Gain- and loss-of-function of UBD are important for the tumorigenesis and progression of CRC. UBD interacts with p53 and promotes p53 degradation through UBD/FAT10ylation of p53, ultimately, promoting CRC occurrence and development (**Figure 8**). There has been many researches confirming that the E1 enzyme UBA6 and E2 enzyme USE1 both are bispecific for UBD and ubiquitin (7, 9, 10). However, the hypothetical E3 enzymes of UBD-mediated proteasome degradation, which remain to be identified. Whether there are other E3 enzymes involved in the effects of UBD on p53 degradation need further investigation. Thus, we found a carcinogenic effect of UBD in CRC, which may act as a valuable prognostic indicator and a potential therapeutic target for CRC.

## Data Availability Statement

The original contributions presented in the study are included in the article/**Supplementary Material**. Further inquiries can be directed to the corresponding author.

## Ethics Statement

The studies involving human participants were reviewed and approved by the Ethics Committee of The First Affiliated Hospital of Chongqing Medical University. The patients/participants provided their written informed consent to participate in this study. The animal study was reviewed and approved by the Ethics Committee of Animal Experiments of Chongqing Medical University.

## Author Contributions

HS and ZX conducted the study design. BJ and XS collected clinical samples. HS and MQ carried out the experiments. HS and QL analyzed and inspected the data. HS drafted the manuscript. ZX reviewed and corrected the manuscript. All authors contributed to the article and approved the submitted version.

## Funding

This study was supported by Natural Science Foundation of Chongqing, China (cstc2019jcyj-msxmX0054).

## Conflict of Interest

The authors declare that the research was conducted in the absence of any commercial or financial relationships that could be construed as a potential conflict of interest.
