# Supplementary material for: Ubiquitin-Like Protein UBD Promotes Cell Proliferation in Colorectal Cancer by Facilitating p53 Degradation
Source: Front Oncol. 2021 Jul 14;11:691347. doi: 10.3389/fonc.2021.691347 (PMC8327751; doi:10.3389/fonc.2021.691347)
Supplement: Supplementary file 1 [file DataSheet_1.pdf]

Supplementary Material

1 Supplementary Tables

Supplementary Table 1. Full description of the antibodies used in this study

Antibody	Catalog number	Dilution ratio	Application	Source	Company
**GAPDH**	10494-1-AP	1:5000	WB	Rabbit	Proteintech Group, Inc. (Proteintech, Wuhan, China)
**p53**	10442-1-AP	1:2000	WB/IP	Rabbit	
**p53**	21891-1-AP	1:200	IHC	Rabbit	
**p21**	10355-1-AP	1:1000	WB	Rabbit	
**CDK6**	14052-1-AP	1:500	WB	Rabbit	
**UBA6**	13211-1-AP	1:1000	WB	Rabbit	
**USE1**	25218-1-AP	1:200	WB	Rabbit	
**MDM2**	27883-1-AP	1:500	WB	Rabbit	
**HRP Goat Anti-Rabbit IgG**	SA00001-2	1:6000	WB	Goat Anti-Rabbit	
**CDK2**	WL01543	1:1000	WB	Rabbit	Wanleibio (Shenyang, China)
**CDK4**	WL02274	1:500	WB	Rabbit	
**Cyclin D1**	WL01435a	1:500	WB	Rabbit	
**Cyclin E**	WL01072	1:500	WB	Rabbit	
**UBD**	ab134077	1:2000	WB	Rabbit	Abcam (Cambridge, UK)
**Ki67**	ab92742	1:500	IHC	Rabbit	
**UBD**	DF7373	1:100	IHC	Rabbit	Affinity Biosciences (Affinity, OH, USA)
**UBD**	sc-393630	1:100	IP	Mouse	Santa Cruz (Dallas, Texas, USA)

Supplementary Table 2. Primers for qPCR

Human Gene Name	Sequence(5'-3')
**UBD**	F: GCTCAGTGGCACAAGTGAAA
	R: GCCGTAATCTGCCATCATCT
**GAPDH**	F: AGAAGGCTGGGGCTCATTTG
	R: AGGGGCCATCCACAGTCTTC

Supplementary Table 3. Human shRNA target sequences

NO.	Target Sequence(5'-3')
**shUBD#1**	GGGATTTAATGACCTTTGA
**shUBD#2**	GGAGCTGCCCTTGTTTCTT
**shp53**	CGGCGCACAGAGGAAGAGAAT
**shUBA6**	GCAAGCAAATCCUGGCATT
**shUSE1**	GGCCTCTGAGGTGATCAAT
**shNC**	TTCTCCGAACGTGTCACGT

2 Supplementary Figure

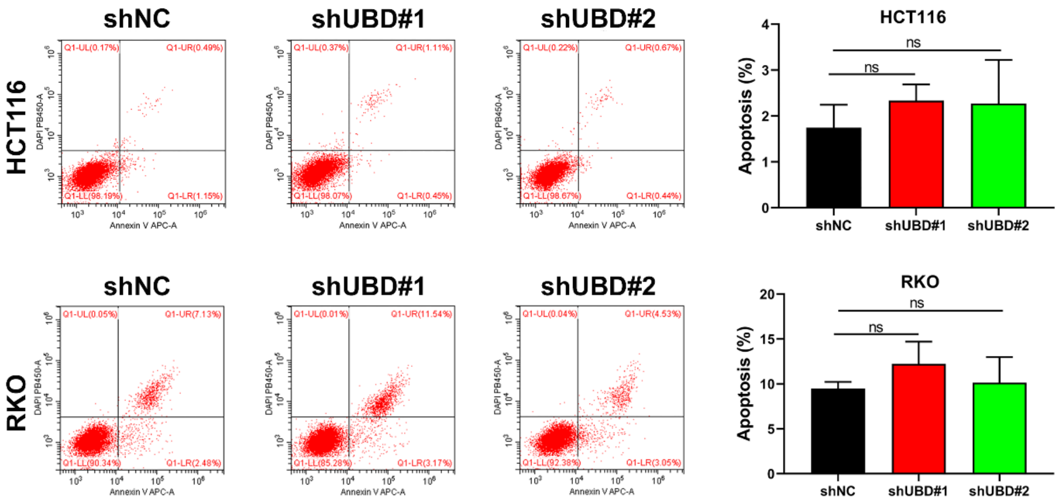

Supplementary Figure 1. Cell apoptosis was analyzed using flow cytometry

Results shown that no significant differences were observed between the shNC group and the UBD stably knockdown group in HCT116 and RKO cells. Revealed that knockdown of UBD had no obviously effect on apoptosis in HCT116 and RKO cells (ns, nonsignificant).

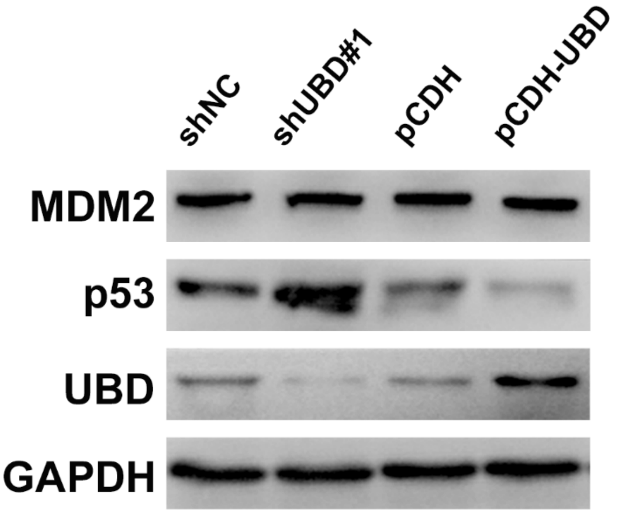

Supplementary Figure 2. Western blotting was used to detected the protein expression of MDM2, p53 and UBD in HCT116 cells transfected with stable knockdown or overexpression plasmid.

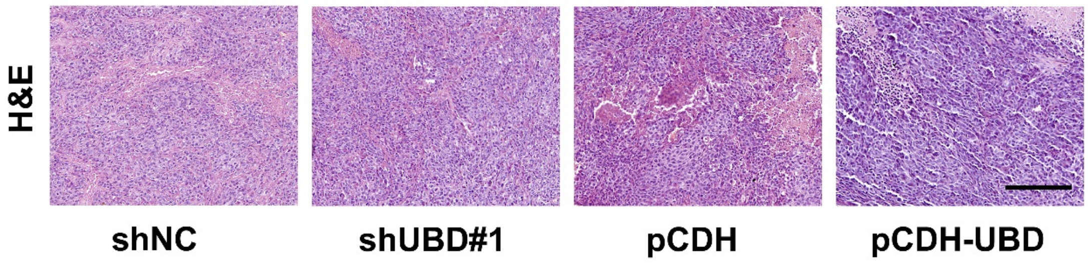

Supplementary Figure 3. H&E staining in xenograft tumor tissues (scale bar, 100 μm)
